# Anal Dilatation

**Published:** 1894-02

**Authors:** 


ANAL DILATATION.—Dilatation of the anus affects more profoundly the respiration, and consequently the capillary circulation, than that of any other of the lower openings of the body. In health nature secures a daily flushing of the capillaries by the passage of large, solid masses of fecal matter. In cases of chronic constipation and chronic diarrhea the system fails to receive its daily benediction of renewed vitality, and consequently suffers from a lack of it. The rectal plugs may be made to act as a substitute for natural dilatation in such cases, and, unaided by other measures, will often restore the habit of the bowel to a normal state, after which their use can be abandoned. The dilators can be used daily in such cases until they have established a satisfactory reaction and the habit becomes normal, or until the irritation which they sometimes induce causes a feeling of pressure at the base of the bladder or local soreness, when their use should be suspended for a time until such irritation has passed away, after which they can be again employed and their action continued until health is restored or they fail to be of further service to the patient.

Rectal dilators may be employed not only by physicians, but may be entrusted to the keeping of intelligent patients to be used at their own discretion. The use of a rectal dilator when one is tired is more invigorating than stimulants. They are also of service in resuscitating a patient from an epileptic seizure or a fainting fit, from opium or other poisoning, and from drowning. They are remarkably efficacious in aiding a new-born infant to its first gasp of breath, and in establishing reaction after prolonged illness, as typhoid fever, diphtheria, pneumonia, gastritis, etc. They are good for a tired brain and a tired body, and are perhaps superior to any other general or remedial agent as an invigorating tonic. In severe cases, such as convulsions, it is well to use the larger size at once, allowing them to remain in position at the discretion of the attending physician.—*Medical Brief.*

